# Predict ovarian cancer by pairing serum miRNAs: Construct of single sample classifiers

**DOI:** 10.3389/fmed.2022.923275

**Published:** 2022-08-02

**Authors:** Guini Hong, Fengyuan Luo, Zhihong Chen, Liyuan Ma, Guiyang Lin, Tong Wu, Na Li, Hao Cai, Tao Hu, Haijian Zhong, You Guo, Hongdong Li

**Affiliations:** ^1^School of Medical Information Engineering, Gannan Medical University, Ganzhou, China; ^2^Medical Big Data and Bioinformatics Research Centre, First Affiliated Hospital of Gannan Medical University, Ganzhou, China

**Keywords:** Serum miRNA, ovarian cancer, early diagnosis, relative expression orderings, single sample classifier

## Abstract

**Objective:**

The accuracy of CA125 or clinical examination in ovarian cancer (OVC) screening is still facing challenges. Serum miRNAs have been considered as promising biomarkers for clinical applications. Here, we propose a single sample classifier (SSC) method based on within-sample relative expression orderings (REOs) of serum miRNAs for OVC diagnosis.

**Methods:**

Based on the stable REOs within 4,965 non-cancer serum samples, we developed the SSC for OVC in the training cohort (GSE106817: OVC = 200, non-cancer = 2,000) by focusing on highly reversed REOs within OVC. The best diagnosis is achieved using a combination of reversed miRNA pairs, considering the largest evaluation index and the lowest number of miRNA pairs possessed according to the voting rule. The SSC was then validated in internal data (GSE106817: OVC = 120, non-cancer = 759) and external data (GSE113486: OVC = 40, non-cancer = 100).

**Results:**

The obtained 13-miRPairs classifier showed high diagnostic accuracy on distinguishing OVC from non-cancer controls in the training set (sensitivity = 98.00%, specificity = 99.60%), which was reproducible in internal data (sensitivity = 98.33%, specificity = 99.21%) and external data (sensitivity = 97.50%, specificity = 100%). Compared with the published models, it stood out in terms of correct positive predictive value (PPV) and negative predictive value (NPV) (PPV = 96.08% and NPV=95.16% in training set, and both above 99% in validation set). In addition, 13-miRPairs demonstrated a classification accuracy of over 97.5% for stage I OVC samples. By integrating other non-OVC serum samples as a control, the obtained 17-miRPairs classifier could distinguish OVC from other cancers (AUC>92% in training and validation set).

**Conclusion:**

The REO-based SSCs performed well in predicting OVC (including early samples) and distinguishing OVC from other cancer types, proving that REOs of serum miRNAs represent a robust and non-invasive biomarker.

## Introduction

Ovarian cancer (OVC) is the most common cancer in female genital organs and is the fifth leading cause of cancer death in females worldwide. The 5-year survival rate for women with localized OVC is 93%, but the rate decreases to 30% for distant OVC, leading to an all-stage combined rate of 49% (1, 2). The strategy for screening OVC commonly relies on clinical transvaginal ultrasound examination and a blood test for the CA125 tumor marker, which is usually performed on women who are at high risk or have symptoms. However, early OVC usually causes no obvious symptoms, and there is a high prevalence of false-positive results of this strategy (1). Therefore, finding sensitive and non-invasive molecular biomarkers that could help detect early OVC is in urgent need.

The discovery of microRNAs (miRNAs), particularly in serum, has opened a new avenue for cancer detection (3). Based on gene expression profiles assayed in high-throughput experiments such as microarray or RNA-seq, many serum miRNA biomarkers have been identified in OVC (4–6). The diagnostic models of these miRNA biomarkers usually rely on a composite score of expression of characteristic genes and classify patients at risk by comparison with pre-defined risk thresholds. However, serum miRNAs can be derived from apoptotic, necrotic, shed cancer cells and other tissue cells or from the secretion of cancer cells, immune leukocytes, etc. (7, 8), and their signals are also affected by changes in the proportional composition of blood leukocytes (9). In general, the signal of serum miRNA expression is relatively weak, which can have an impact on cancer discrimination and the specificity of serum-based biomarkers. Moreover, miRNA expression levels are susceptible to batch effects, individual genetics, and technical fluctuations (10). Therefore, preprocessing like standardization of data is required when applying such biomarkers, which makes these biomarkers difficult to apply to individual clinical practice (11).

Considering the different preprocessing requirements of biomarkers, a new type of biomarkers has emerged, namely the single sample classifier (SSC) (12). The decision rule of SSC is based on the within-sample relative expression orderings (REOs) between two genes, which can be interpreted as if the expression of gene A is smaller than the expression of gene B, the sample is assigned to class *C*; otherwise, it is non-*C* class. The underlying assumption of REO-based SSC is that, under normal conditions, although external environmental stimuli can affect gene expression in the organism and its cells, the affected genes should normally exhibit coordinated biological activity, behaving as the REOs of most genes should be in a stable relative equilibrium. Studies have demonstrated this biological coordination phenomenon, whereby REOs of genes are broadly stable in normal samples and altered when a disease such as cancer occurs (10, 13). More importantly, REOs have the unique advantage of being insensitive to batch effects, data normalization methods, partial RNA degradation, RNA amplification bias, and the proportion of different cancer epithelial cells (14, 15). REO-based SSCs can therefore be used as diagnostic biomarkers for cancer and are particularly suitable for individual clinical diagnosis.

In this study, we aimed to construct an REO-based single sample serum miRNA classifier and compare it with traditional risk scoring models constructed based on the expression combination of single miRNAs. An SSC consisting of 13 miRNA pairs was developed, using OVC as the context, involving a total of 8,184 samples comprised of 360 high-grade plasma OVC and 7,824 non-cancer control samples. This classifier showed comparable sensitivity and specificity and stood out in terms of correct positive predictive value (PPV) and negative predictive value (NPV) compared to the published models (4, 6). Moreover, it achieved high classification accuracy for stage I OVC samples, demonstrating its potential as a diagnostic biomarker for early-stage OVC. Finally, we analyzed the expression and biological function of the selected serum miRNAs. An alternative classifier consisting of 17 miRNA pairs was also developed to distinguish OVC from 1,339 other types of cancer samples by integrating other non-OVC serum samples together as controls.

## Materials and methods

### Data sources

A total of 9,523 serum samples from three datasets were analyzed (Table 1), with pre-processed miRNA expression values and their clinical data downloaded from Gene Expression Omnibus (GEO; https://www.ncbi.nlm.nih.gov/geo/). GSE122497 dataset included 566 esophageal cancer and 4,965 non-cancer samples, and only the non-cancer control samples were analyzed in the study. In GSE106817, the 320 high-grade serous OVC samples and 2,759 non-cancer control samples were randomly split into two sets: a training and a test set. The training set contained 200 OVCs as cases and 2,000 non-cancer samples as controls, while the test set contained 120 OVCs as cases and 759 non-cancer samples as controls. The GSE113486 dataset served as the validation set, containing 40 OVCs as cases and 100 healthy control samples. The GSE106817 dataset and GSE113486 also had 859 and 832 non-OVCs of various cancer types, respectively. In particular, we randomly took 40 samples from the 392 bladder cancer samples to maintain a sample size of 40 in line with the other 11 cancer types in GSE113486. The non-OVC cancer samples in these two datasets were used as controls for developing and validating the ovarian-specific SSC, respectively.

**Table 1:** The datasets and samples analyzed in the study.

**Dataset^a^**	**GEO accession**	**Non-cancer (N)**	**Cancer(N)**	**Ref**
Training	GSE122497	4,965	-	(16)
	GSE106817	2,000	Ovarian: 200; Breast: 115; Colorectal: 115; Esophageal: 88; Gastric: 115; Hepatocellular Carcinoma: 81; Lung: 115; Pancreatic: 115; Sarcoma: 115	(6)
Test	GSE106817	759	Ovarian: 120	(6)
Validation	GSE113486	100	Ovarian:40; Breast: 40; Colorectal: 40; Esophageal: 40; Gastric: 40; Hepatocellular Carcinoma: 40; Lung: 40; Pancreatic: 40; Sarcoma: 40; Biliary Tract: 40; Bladder: 40; Glioma: 40; Prostate: 40	(17)

^a^*Serum samples were profiled by 3D-Gene Human miRNA V21_1.0.0*.

### Definition of within-sample relative expression orderings of miRNAs

For a miRNA dataset, the expression profiles can be represented as a matrix *E* with dimension *M* × *N*, where *M* represents the number of assayed miRNAs and *N* represents the number of profiles (samples). A profile either belongs to class *C* (cancer samples) or non-C class (control samples) and could be denoted as [*E*_1_, …, *E*_*i*_, …, *E*_*M*_], where *E*_*i*_ represents the expression level for miRNA *i*. Let *E*_*a*_ denote the expression value of miRNA *a*, and *E*_*b*_ denote the expression value of miRNA *b*. Then, the within-sample REO of two miRNAs, *a* and *b*, is defined as the relatively bigger or smaller expression relationship between them, denoted as *E*_*a*_ > *E*_*b*_ or *E*_*a*_<*E*_*b*_, depending on the expression values of *E*_*a*_ and *E*_*b*_.

### Definition of stable miRNA pairs and reversed miRNA pairs

In a miRNA expression profile, any two miRNAs can form a miRNA pair. If *n* miRNAs are assayed, there could be *n* (*n*-1)/2 miRNA pairs.

Stable miRNA pairs were identified from a large cohort of *N* non-cancer samples. Assuming that *E*_*a*_ > *E*_*b*_ is observed in *m* of the *N* non-cancer samples for a miRNA pair (*a, b*), the probability can be expressed as *P* (*E*_*a*_ > *E*_*b*_) = *m/N*. If *P* (*E*_*a*_ > *E*_*b*_) is greater than a threshold, for example, 95%, then the miRNA pair (*a, b*) is defined as a stable miRNA pair.

For the training set, only stable miRNA pairs that maintain their REOs in more than a high proportion (e.g., 95%) of non-cancer training samples are retained for detection of reversed miRNA pairs. Then, for each retained stable miRNA pair, the numbers of non-cancer controls with *E*_*a*_ > *E*_*b*_ and *E*_*a*_<*E*_*b*_ were calculated and denoted by *n*_1_ and *n*_2_, and the numbers of cancer samples with *E*_*a*_ > *E*_*b*_ and *E*_*a*_<*E*_*b*_ were calculated and denoted by *m*_1_ and *m*_2_, respectively. According to *n*_1_, *n*_2_, *m*_1_, and *m*_2_, we used Fisher's exact test to assess whether the REO of a stable miRNA pair in non-cancer controls was significantly reversed in cancer samples.

### The design of the diagnostic model

The diagnostic model is constructed as described below.

(1) Select the candidate diagnostic miRNA pairs. For a significantly reversed miRNA pair, the higher the reversal proportion in a cancer sample, the more predictive potential the pair possesses. For a set of significantly reversed miRNA pairs (denoted as *S*), the reversal proportion of covering cancer samples was calculated by *k*% as follows: ∀(*a, b*) ∈ *S* (*a* ≠ *b*), *E*_*a*_<*E*_*b*_ holds in at least *k*% instances among cancer samples.
(2) Search a combination (denoted as *C*) for each candidate diagnostic miRNA pair with the biggest percent of joint covering samples with the smallest number of pairs. Here the percent of joint covering cancer (or non-cancer) samples for *C* was calculated by *p* = *k*/*N*_1_ (or *k*/*N*_2_), where *k* denotes the number of samples whose REO present as *E*_*a*_<*E*_*b*_ (or *E*_*a*_ > *E*_*b*_), ∀(*a, b*)∈*C* (*a* ≠ *b*), and *N*_1_ (or *N*_2_) denotes the number of cancer (or non-cancer) samples. For example, for a pair (*a, b*) in the list of candidate diagnostic miRNA pairs (denoted as *D*), at first, *C*_(*a, b*)_ = {(*a, b*)}. Then, a miRNA pair (*c, d*) ∈*D* (*c* ≠ *d*) is added to *C*_(*a, b*)_ such that *C*_(*a, b*)_={(*a, b*), (*c, d*)}, as that the percent of joint covering samples of *C*_(*a, b*)_={(*a, b*), (*c, d*)} was greater than that of *C*_(*a, b*)_={(*a, b*)} and that of *C*_(*a, b*)_={(*a, b*), (*g, h*)}, ∀(*g, h*)∈*D*. The procedure of adding miRNA pairs from the remaining was stopped until there was no further increase in the percent of joint covering cancer samples of *C*_(*a, b*)_.
(3) Count the frequency of occurrence of each candidate miRNA pair in all combinations and rank in descending order of frequency of occurrence.
(4) The top *n* (*n* is odd) miRNA pairs in (3) are selected to classify the samples in the training set. The criterion for classification is the voting rule: for a sample to be classified when more than half of the top *n* miRNA pairs consisting of one to *n* miRNA pairs hold *E*_*a*_<*E*_*b*_, the sample is judged to be a cancer sample; otherwise, the sample is judged to be a non-cancer control.
(5) Calculate the classification evaluation index, namely the square root of the product of PPV and NPV. The top *n* miRNA pairs corresponding to the highest evaluation index were selected as the final diagnostic classifier.

### Target analysis of diagnostic miRNAs

The miRNA target prediction tool microRNA Data Integration Portal (miRDIP) (http://ophid.utoronto.ca/mirDIP) integrates 30 different resources of human miRNA-target prediction tools to integrate all data related to miRNA-target interactions (18). We used it (Version 5.0.2.3, June 2021) to assess the targets of miRNAs involved in our diagnostic model.

### Statistical analyses

Statistical analyses were performed using R version 3.5.2 (https://cran.rproject.org/). Differential miRNA expression analysis was performed between case and control samples using an unpaired *t*-test. Wilcoxon rank-sum test was used to compare the mean and variance of expression. Fisher's exact test was used to evaluate whether the REOs of the stable miRNA pairs are significantly reversal in cancer samples. The functional enrichment analysis was performed using KEGG pathways by the R package “clusterProfiler” with default parameters. Multiple testing adjusted *p*-values (i.e., false discovery rate *q* values) were computed using Benjamini-Hochberg (BH) method (19). A *q*-value smaller than 0.05 was considered significant. Diagnostic accuracy, sensitivity, specificity, PPV and NPV, and area under the receiver operating characteristic curve (AUC) were calculated for the diagnostic model.

## Results

### The 13-miRPairs serum single sample classifier

**Figure 1:**
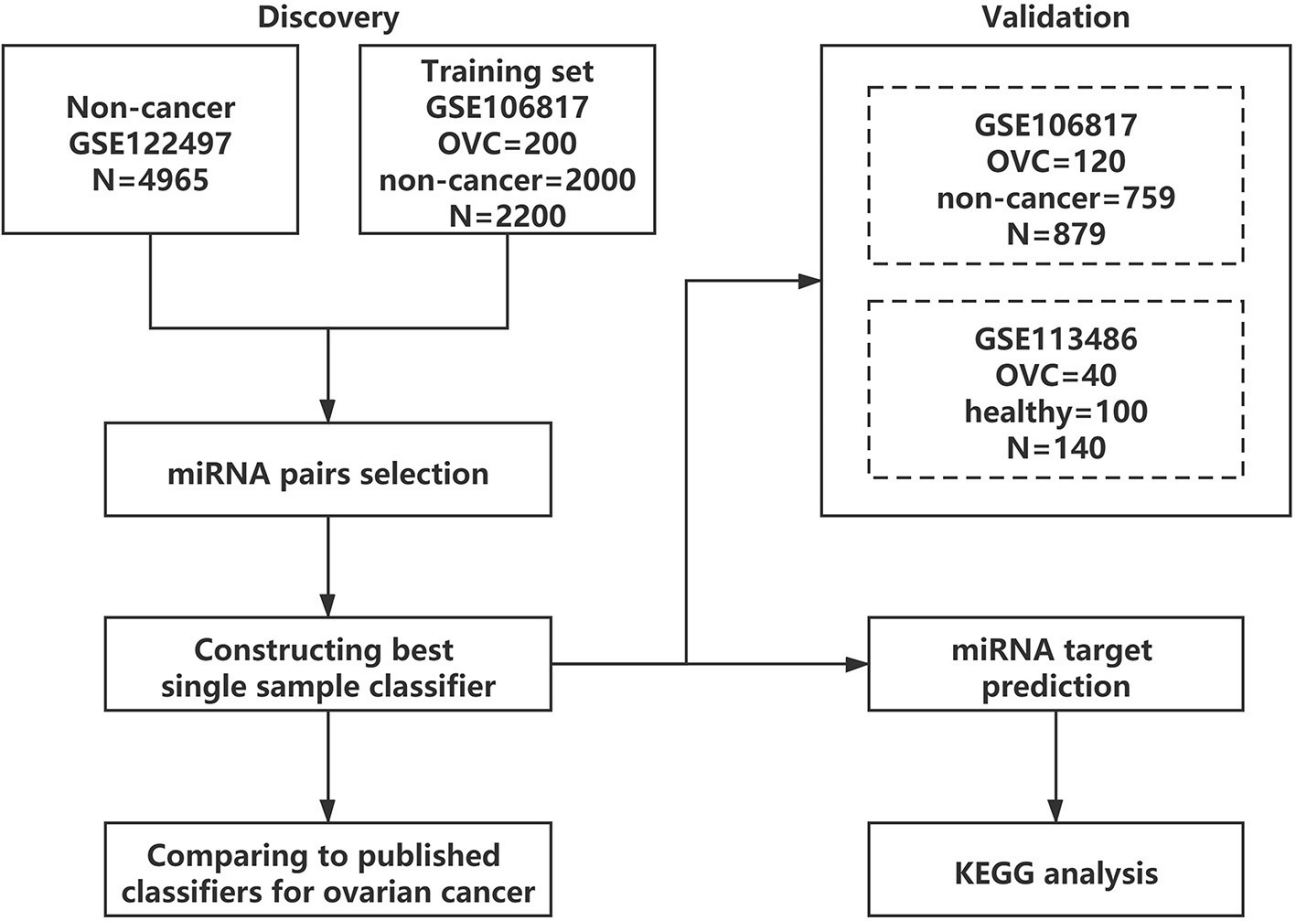
Overall flowchart.

**Figure 2:**
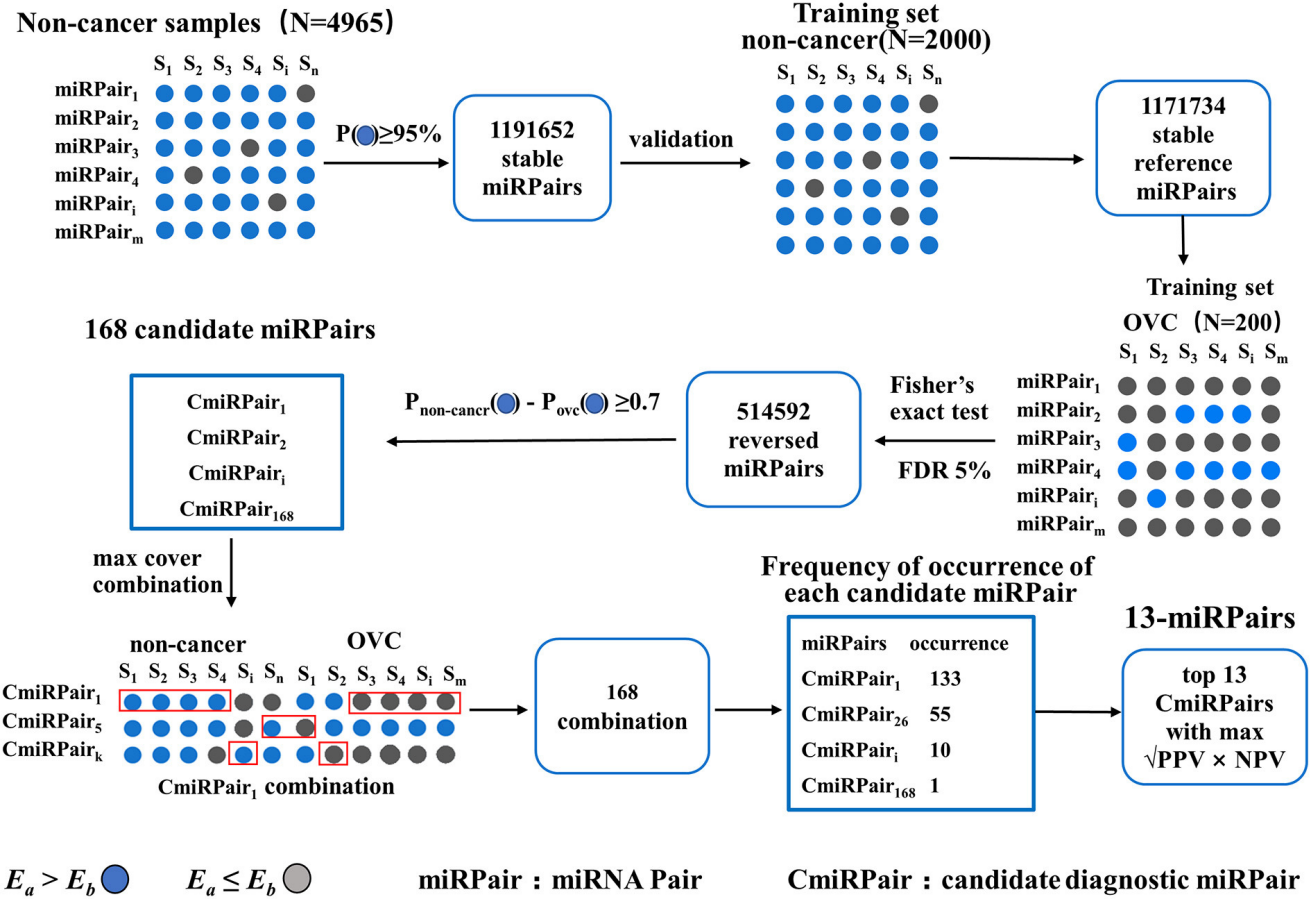
Steps of constructing 13-miRPairs and their detailed results.

An outline of the study design is presented in Figure 1. First, the serum diagnostic single sample classifier for detecting OVC was constructed, and the detailed results are described below and illustrated in Figure 2.

The first step is to identify miRNA pairs that have stable REOs in the large cohort of 4,965 non-cancer serum samples. A miRNA pair is defined as a stable pair if it maintains its REO pattern in a certain percentage of control samples (see Methods). Let the percentage be 95%. We obtained 1,191,652 stable miRNA pairs. Among these stable miRNA pairs, 1,171,734 (98.33%) kept their stable REOs in 2,000 non-cancer control samples in the training set, which were used as reference miRNA pairs for subsequent analysis.

**Figure 3:**
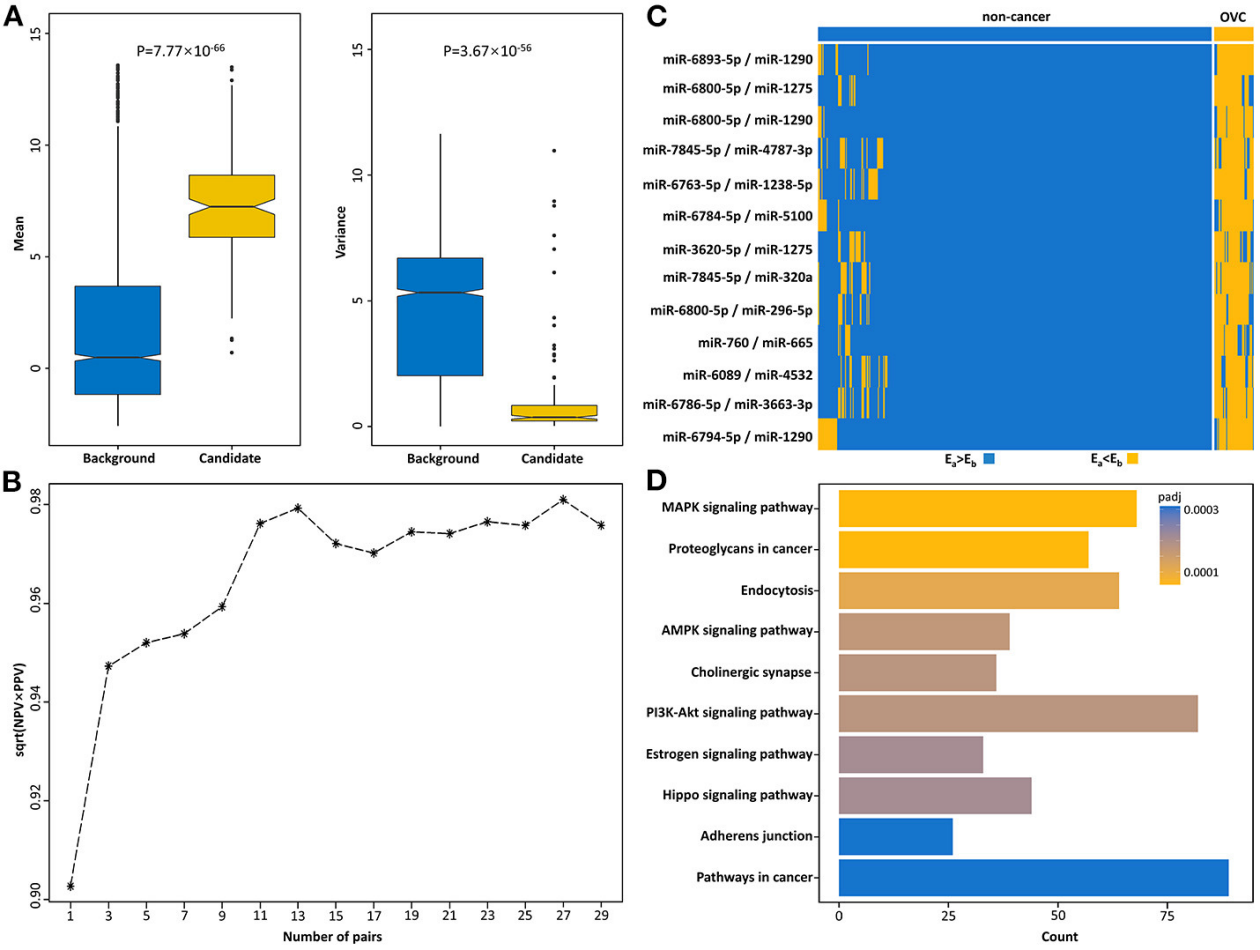
Identification of serum diagnostic miRNA pairs for OVC. **(A)** Boxplots of mean and variance of expression of miRNAs in background and candidate diagnostic miRNA pairs. **(B)** The square root of PPV and NPV of candidate top miRNA pairs for diagnosing OVC in the training set. **(C)** The corresponding REOs of 13-miRPairs. **(D)** The 13-miRPairs associated KEGG pathways.

Then, miRNA pairs whose REOs were significantly reversed under the OVC condition were identified from 200 OVC samples in the training set based on the reference miRNA pairs. With FDR <0.05, 514,592 significantly reversed miRNA pairs were determined. Then, candidate diagnostic miRNA pairs were determined from the significantly reversed miRNA pairs. If a reversed miRNA pair showed *E*_*a*_<*E*_*b*_ in more than 70% of OVC samples, it was selected as a candidate diagnostic miRNA pair. Totally, we obtained 168 candidates. In terms of expression abundance, those miRNAs involved in these candidate diagnostic miRNA pairs had significantly higher expression levels than the background miRNAs (rank-sum test, *P* = 7.77 ×10^−66^, Figure 3A). At the same time, the variance was much smaller than that of the background miRNAs (*P* = 3.67 × 10^−56^, Figure 3A). The results indicate the potential of these miRNA pairs as candidate diagnostic biomarkers.

The next step was to find the most predictive pairs from the candidate diagnostic miRNA pairs. Briefly, the procedure includes three steps (see Methods and Figure 2). First, each of the 168 candidate miRNA pairs was used as a pivot, and the other miRNA pairs beside the pivot were used to compensate for their coverage of samples to form a combination that covered the largest number of samples. Second, among the 168 combinations formed, the frequencies of occurrence of the reversed miRNA pairs were counted. By sorting the frequency of occurrence from highest to lowest, the 31 miRNA pairs with the highest frequencies were selected. Third, comprehensive combinations consisting of one to 31 top miRNA pairs were selected, and the square root of (PPV × NPV) was calculated as the evaluation index from the training set according to the voting rule. It should be noted that each combination of the top miRNA pairs had good diagnostic potential, all with an evaluation index above 90%. For example, when the top three miRNA pairs were selected as the diagnostic classifier, the evaluation index was 94.7%. Finally, by choosing the combination with the largest evaluation index and the lowest number of miRNA pairs, we obtained a combination comprised of the top 13 miRNA pairs as the best classifier in the training set (√PPV × NPV = 0.979, Figure 3B), referred to as 13-miRPairs, which involves a total of 20 miRNAs (Table 2).

**Table 2:** The 13-miRPairs classifier developed for distinguishing OVC from non-cancer samples.

**miRNA *a***	**miRNA *b***	**Non-cancer (%)** ***E_*a*_* > *E_*b*_***	**OVC (%) *E_*a*_* > *E_*b*_***	***P*-value^a^**
miR-6893-5p	miR-1290	0.981	0.095	<2.2 ×10^−16^
miR-6800-5p	miR-1275	0.977	0.190	<2.2 ×10^−16^
miR-6800-5p	miR-1290	0.987	0.120	<2.2 ×10^−16^
miR-7845-5p	miR-4787-3p	0.954	0.160	<2.2 ×10^−16^
miR-6763-5p	miR-1238-5p	0.951	0.070	<2.2 ×10^−16^
miR-6784-5p	miR-5100	0.977	0.110	<2.2 ×10^−16^
miR-3620-5p	miR-1275	0.965	0.260	<2.2 ×10^−16^
miR-7845-5p	miR-320a	0.965	0.095	<2.2 ×10^−16^
miR-6800-5p	miR-296-5p	0.974	0.165	<2.2 ×10^−16^
miR-760	miR-665	0.980	0.285	<2.2 ×10^−16^
miR-6089	miR-4532	0.959	0.225	<2.2 ×10^−16^
miR-6786-5p	miR-3663-3p	0.956	0.115	<2.2 ×10^−16^
miR-6794-5p	miR-1290	0.951	0.130	<2.2 ×10^−16^

^a^*Fisher's exact test; OVC, ovarian cancer*.

### Diagnostic performance of 13-miRPairs

In the training set, the sensitivity and specificity of the 13-miRPairs classifier were 98.00% and 99.60%, respectively. When applied to the 759 non-cancer and 120 OVC samples in the test set, the sensitivity and specificity were 98.33% and 99.21%, respectively. In the validation set comprised of 100 healthy and 40 OVC samples, the sensitivity dropped slightly to 97.50%, but the specificity reached 100%. In addition to sensitivity and specificity, it may be more important for clinicians and patients to consider the PPV and NPV of a diagnostic signature. Therefore, we also evaluated the PPV and NPV of the 13-miRPairs classifier. As shown in Table 3, the PPV and NPV remained high at 96.08% and 99.80% in the training set, respectively, and 95.16% and 99.74% in the test set, respectively. In the validation set, 13-miRPairs had more than 99% diagnostic performance in both evaluation indexes (PPV = 100%, NPV = 99.01%). The above results indicate a good diagnostic performance of the 13-miRPairs classifier.

**Table 3:** Diagnostic performance of 13-miRPairs, 10-miRNAs, and OCaMIR.

**Dataset: model**	**SEN%**	**SPE%**	**ACC%**	**PPV%**	**NPV%**
Training: 13-miRPairs	98.00	99.60	99.45	96.08	99.80
Training: 10-miRNA	99.00	98.95	98.95	90.41	99.90
Test: 13-miRPairs	98.33	99.21	99.09	95.16	99.74
Test: 10-miNRA	1.00	98.16	98.41	89.55	1.00
Validation: 13-miRPairs	97.50	1.00	99.29	1.00	99.01
Validation: 10-miRNA	97.50	1.00	99.29	1.00	99.01
^a^Original training: OCaMIR	88.44	73.75	81.09	77.11	86.45
^a^Original validation: OCaMIR	84.62	75.27	78.03	58.93	92.11

^a^*Results were extracted originally from Usuba et al. by applying to their training data set (GSE106817 without sample partition) and test data set (GSE113486). ACC, accuracy; SEN, sensitivity; SPE, specificity; PPV, positive predictive value; NPV, negative predictive value*.

### Comparing the performance of 13-miRPairs with published OVC diagnostic models

Yokoi et al. constructed a diagnostic model containing 10 miRNAs (referred to as the 10-miRNA model) with high sensitivity (100% and 99%) and specificity (100% and 100%) in their training and validation set for discriminating OVC and non-cancer samples, respectively (6). The diagnostic model relies on the expression of these 10 miRNAs: diagnostic index = (0.581) × miR-320a + (0.691) × miR-665 + (−0.704) × miR-3184-5p + (−0.313) × miR-6717-5p + (−1.302) × miR-4459 + (0.729) × miR-6076 + (0.676) × miR-3195 + (0.716) × miR-1275 + (0.672) × miR-3185 + (-0.384) × miR-4640-5p - 9.375 (<0, non-cancer; ≥0, OVC). We reproduced the 10-miRNA model and applied it to our partition of the 3,079 sample. The results showed that the 10-miRNA model maintained ~99% diagnostic performance in both our training and test set in terms of sensitivity and specificity (99% and 100% for sensitivity and 98.95% and 98.16% for specificity), respectively (Table 3). However, the PPV of 10-miRNA was only 90.41% and 89.55% on both the training and test set, significantly lower than the PPV (96.08% and 95.16%) of the 13-miRPairs classifier. For the validation set, the classification ability of the two models was the same, both above 99%.

The OCaMIR model constructed by Kandimalla et al. consists of 8 miRNAs, with a sensitivity and specificity of 88.44% and 73.75%, and a PPV and NPV of 77.11% and 86.45%, for differentiating OVC and healthy samples in their training set (4). In their validation set of GSE113486, the sensitivity and specificity are 84.62% and 75.27%, and the PPV and NPV are 58.93% and 92.11%, respectively. Compared with the OCaMIR model, our 13-miRPairs classifier has much higher diagnostic performance: as shown in Table 3, the sensitivity, specificity, PPV, and NPV were 97.50%, 100%, 100%, and 99.01% on the same data set, respectively. Notably, for the 81 stage I OVC samples in GSE106817, only 54% were predicted as positive by the OCaMIR model designed for early-stage OVC detection (4). At the same time, our 13-miRPairs classifier classified 97.5% of stage I patients as OVC, indicating that 13-miRPairs is more suitable for early detection.

The above results indicate that the combination of the 13-miRPairs classifier represents a promising signature for OVC screening.

### Expression and functional characterizations of the diagnostic miRNAs

All 20 miRNAs in the 13-miRPairs classifier were differentially expressed in OVC samples compared to non-cancer controls, with ten up-regulated and ten down-regulated (*t*-test, q-value <0.05). Their REOs in the training set were also displayed by a heat map (Figure 3C). It is easy to visualize how these miRNA pairs can classify the samples. For a sample to be classified, if all 13 miRNA pairs exhibit *E*_*a*_ > *E*_*b*_ (or *E*_*a*_<*E*_*b*_), that is, all blue (or yellow) in the heat map, it is judged to be a non-cancer (or OVC) sample. If some miRNA pairs exhibit *E*_*a*_ > *E*_*b*_, then the label is determined with the majority voting technique. That is, if more miRNA pairs show *E*_*a*_ > *E*_*b*_, then it is judged as a non-cancer sample, otherwise as an OVC sample.

Many of the deregulated miRNAs have been reported to be associated with the growth and progression of OVC. For example, downregulation of the miR-760 has been reported to inhibit the proliferation of OVC cells (20). Liu et al. reported that miR-6089 serves as a tumor suppressor with its overexpression suppressing the proliferation, migration, invasion, and metastasis of OVC, while in fresh ovarian tissue, it is downregulated compared to paracancerous tissue (21), which is consistent with our results.

KEGG analysis showed that the mRNA targets of the 13-miRPairs classifier were significantly enriched in many cancer-associated pathways (Figure 3D). Among the most significant 10 pathways, five were signal transduction pathways, including the MAPK signaling pathway, AMPK signaling pathway, PI3K-Akt signaling pathway, Estrogen signaling pathway, and Hippo signaling pathway. In particular, the PI3K/AKT/mTOR cascade has been identified as frequently altered in OVC (22). The three signaling pathways were all significantly disturbed by the diagnostic miRNAs, implying their important role in OVC.

### Distinguishing OVC from other cancer types

Next, we applied 13-miRPairs to other cancers. As shown in Table 4, more than 60% of different cancer samples were predicted as positive, except for breast cancer. These results implied that it might be challenging to determine ovarian cancer from other cancer types for 13-miRPairs, although it can distinguish ovarian cancer from non-cancer samples.

**Table 4:** Diagnosis of multiple cancer types by the 13-miRPairs classifier.

**Data**	**GSE106817**		**GSE113486**	
	**Sample No. (*N*)**	**Proportion^a^**	**Sample No. *(N)***	**Proportion**
Breast cancer	115	0.87%	40	7.50%
Colorectal cancer	115	88.70%	40	75.00%
Esophageal cancer	88	98.86%	40	100%
Gastric cancer	115	96.52%	40	0.8750
Hepatocellular carcinoma	81	91.36%	40	92.50%
Lung cancer	115	98.26%	40	97.50%
Pancreatic cancer	115	96.52%	40	87.50%
Sarcoma	115	75.65%	40	60.00%
Biliary tract cancer	-	-	40	97.50%
Bladder cancer	-	-	40	97.50%
Glioma	-	-	40	90.00%
Prostate cancer	-	-	40	82.50%

^a^*The proportion of cancer samples predicted to be ovarian cancer by 13-miRPairs*.

To obtain an OVC-specific serum SSC, we developed another classifier, training with 320 OVCs as the cases and 859 samples from eight other cancers in the GSE106817 dataset as controls. The OVC-specific classifier consisted of 17 top miRNA pairs (Table 5), with an AUC of 0.9581 (sensitivity = 85%, specificity = 91.15%) for classifying ovarian and other cancers in the training set. The 17-miRPairs classifier also achieved an AUC of 0.9205 (sensitivity = 82.50%, specificity = 85.62%) for classification when applied to the 40 OVC samples and 480 samples of 12 other types of cancers in the validation dataset GSE113486. This confirms the high specificity of the REO-based SSC of serum miRNAs for detecting OVC.

**Table 5:** The 17-miRPairs classifier developed for distinguishing OVC from other cancers.

**miRNA *a***	**miRNA *b***	**Non-OVC (%)** ***E_*a*_* > *E_*b*_***	**OVC (%) *E_*a*_* > *E_*b*_***	***P*-value^a^**
miR-6746-5p	miR-6887-5p	0.7858	0.3938	<2.2 ×10^−16^
miR-6794-5p	miR-6741-5p	0.9162	0.5469	<2.2 ×10^−16^
miR-1343-3p	miR-6741-5p	0.7695	0.3000	<2.2 ×10^−16^
miR-211-3p	miR-1249-5p	0.8161	0.2688	<2.2 ×10^−16^
miR-6717-5p	miR-6887-5p	0.7520	0.2344	<2.2 ×10^−16^
miR-211-3p	miR-665	0.7066	0.1906	<2.2 ×10^−16^
miR-650	miR-6736-5p	0.7206	0.2938	<2.2 ×10^−16^
miR-6800-5p	miR-642a-3p	0.7066	0.3063	<2.2 ×10^−16^
miR-6748-5p	miR-6736-5p	0.7567	0.3969	<2.2 ×10^−16^
miR-6746-5p	miR-7114-5p	0.7101	0.2750	<2.2 ×10^−16^
miR-711	miR-1249-5p	0.8801	0.5156	<2.2 ×10^−16^
miR-939-5p	miR-8071	0.7392	0.3813	<2.2 ×10^−16^
miR-6877-5p	miR-6741-5p	0.7101	0.3219	<2.2 ×10^−16^
miR-1224-5p	miR-6736-5p	0.7299	0.3563	<2.2 ×10^−16^
miR-760	miR-6779-5p	0.7509	0.4406	<2.2 ×10^−16^
miR-6769a-5p	miR-7114-5p	0.7590	0.3188	<2.2 ×10^−16^
miR-3162-5p	miR-6124	0.7078	0.2531	<2.2 ×10^−16^

^a^*Fisher's exact test*.

## Discussion

Currently, the exploration and discovery of diagnostic biomarkers with clinical translational value are one of the important tasks in OVC-related research (23). Although many diagnostic models based on serum miRNA expression have been developed for OVC, these models often rely on pre-determined risk thresholds. However, weak signals and inter-individual variation in serum miRNA expression can exacerbate the problem of setting risk thresholds and impede the clinical application of such biomarkers (24, 25). In this study, we developed an accurate and non-invasive new method for detecting OVC, a single sample classifier based on the REOs of serum miRNAs. Our classifier has many advantages compared to diagnostic models constructed based on single serum miRNAs. Firstly, the REOs of serum miRNAs are not as susceptible to technical fluctuations, batch effects, and data normalization methods as expression levels. Thus, the REO-based SSCs are highly reproducible in independent data. Secondly, the expression levels of some miRNAs are subject to individual differences and show large fluctuations between individual cancer patients. Traditional OVC diagnostic biomarkers constructed using combinations of expression levels of single miRNAs have difficulty coping with such fluctuations. In contrast, REO-based SSCs are only associated with relative changes within individual samples and not with other samples, and do not suffer from the effects of individual fluctuations. Finally, the goals pursued in clinical practice are ease of use and better diagnostic performance of biomarkers. Our classifier outperforms published diagnostic models of single serum miRNAs, demonstrating its high accuracy, non-invasiveness, and clinical translational value.

The first SSC developed in this study, the 13-miRPairs classifier, corresponds to a common clinical diagnosis requirement: distinguishing between OVC and non-cancer samples. For this diagnostic scenario, our control sample settings in the training set are different from the settings of the OCaMIR model developed by Kandimalla et al. (4). The control samples we used were non-cancer samples, including healthy individuals and benign patients without cancer. In contrast, the training control samples for OCaMIR were only healthy, which may not be clinically practical. A common clinical scenario is that patients come one after the other and are relatively rarely completely healthy, for example, often suffering from inflammatory or other benign conditions. Intuitively, when the OCaMIR model is applied in clinical practice, it may judge non-cancer samples as cancerous. By further analysis, our results showed that the OCaMIR model did have low diagnostic efficacy in our training and validation samples, with sensitivity and PPV both below 40%, respectively. On the other hand, we extracted 320 healthy samples from the non-cancer samples in GSE106817 and applied the 13-miRPairs classifier to make predictions. The results showed that the prediction accuracy of these healthy samples was 98.125%. Therefore, our setting of the control population is more suitable for clinical application scenarios of cancer detection.

Notably, a pre-printed article trains serum samples of 13 cancers (including OVC) and non-cancer serum samples and identifies a set of serum pan-cancer biomarkers consisting of five miRNAs (26). We found that one of the selected miRNAs, miR-6784-5p, is also included in our 13-miRPairs classifier, indicating the pan-cancer predictive ability of this miRNA. Because CA125 is not a specific marker for OVC, its elevated level may also indicate the risk of pancreatic, lung, and breast cancer. Therefore, besides the distinction mentioned above in clinical practice between OVC and non-cancer, another common requirement is to distinguish OVC from other cancers. In response to this need, we have developed another diagnostic model, the 17-miRPairs classifier model. For the 12 cancer types in the independent validation set, this model performs well at distinguishing OVC from other cancers, proving the clinical diagnostic value of the REO-based SSCs.

There are also some limitations in this study. First, the miRNA serum expression profiles used in the study were all from the Japanese population and were detected by the same miRNA detection platform (the 3D-Gene® human miRNA platform V21). Secondly, there was also an imbalance between the number of cancer and non-cancer samples in constructing the classifier, which may lead to the under-representation of cancer samples. In addition, further validation in a clinical setting is needed for translational applications.

## Data availability statement

The datasets presented in this study can be found in online repositories. The names of the repository/repositories and accession number(s) can be found in the article/supplementary material.

## Author contributions

GH and HL conceived the idea and conceptualized the study. GH conducted the bioinformatics analysis, interpreted results, and wrote the paper. LM, GL, TW, NL, HC, and TH collected and pre-processed data. FL and ZC generated the figures and tables. HL supervised the whole study process. HZ helped to revise the manuscript. HL, YG, and GH revised the manuscript. All authors have read and approved the final version of manuscript.

## Funding

This work was supported in part by the National Natural Science Foundation of China (Grant No. 61961002), the Doctoral Fund of Gannan Medical University (Grant Nos. QD201827, and QD201828), and the Thousand Talents Program of Jiangxi for High-Level Talents in Innovation and Entrepreneurship (with HL, No. Jxsq2020101096).

## Conflict of interest

The authors declare that the research was conducted in the absence of any commercial or financial relationships that could be construed as a potential conflict of interest.

## Publisher's note

All claims expressed in this article are solely those of the authors and do not necessarily represent those of their affiliated organizations, or those of the publisher, the editors and the reviewers. Any product that may be evaluated in this article, or claim that may be made by its manufacturer, is not guaranteed or endorsed by the publisher.
